# Mortality in COPD: Inevitable or Preventable? Insights from the Cardiovascular Arena

**DOI:** 10.1080/15412550802093041

**Published:** 2008-06-13

**Authors:** David Halpin

**Keywords:** Chronic obstructive pulmonary disease, COPD, mortality, inhaled corticosteroids, long-acting β_2_-agonists, cardiovascular disease

## Abstract

Mortality due to chronic obstructive pulmonary disease continues to rise, whereas mortality rates related to cardiovascular disease appear to be slowing, or even declining. This is due at least in part to more widespread use of preventative therapies that have been shown to reduce cardiovascular mortality, raising the question of whether appropriate use of therapies for chronic obstructive pulmonary disease which potentially reduce mortality could have a similar impact. This article discusses approaches used successfully in managing heart disease and considers whether these can be applied to chronic obstructive pulmonary disease and whether a better understanding of the strongest predictors of mortality in chronic obstructive pulmonary disease is needed. It reviews the role of inhaled corticosteroids, both alone and in combination with long-acting β_2_-agonists, in individuals with chronic obstructive pulmonary disease, including the role of combination therapy with inhaled corticosteroids/long-acting β_2_-agonists (budesonide/formoterol or salmeterol/fluticasone propionate) in decreasing exacerbations and improving health status, potentially providing survival benefits in chronic obstructive pulmonary disease. This review also discusses the potential impact of treatments indicated for cardiovascular disease on chronic obstructive pulmonary disease and possible links between the two diseases.

## INTRODUCTION

Chronic obstructive pulmonary disease (COPD) is defined by the Global Initiative for Chronic Obstructive Lung Disease (GOLD) 2006 Guidelines as “*a preventable and treatable disease with some significant extrapulmonary effects that may contribute to the severity in individual patients. Its pulmonary component is characterized by airflow limitation that is not fully reversible. The airflow limitation is usually progressive and associated with an abnormal inflammatory response of the lung to noxious particles or gases*” (1). COPD is usually caused by cumulative exposure to tobacco smoke, which in the western world is the dominant factor in up to 90% of cases, but occupational dusts and chemicals, and indoor or outdoor air pollution may also play a role (1). In addition to the pulmonary manifestations of the disease, COPD is also associated with systemic effects that may lead to osteoporosis, skeletal muscle dysfunction and cachexia (1, 2).

COPD frequently coexists with other chronic conditions and the presence of these co-morbidities adversely affects outcome. Some of the co-morbidities share a common aetiology with COPD and in the past it has been assumed that this explained their coexistence. Recently, however, it has been recognized that additional systemic effects of COPD include an increased risk of myocardial infarction (MI) and other cardiovascular manifestations, most of which are likely to be associated with an ongoing low-grade systemic inflammation (2).

COPD is a common disease. In 2002, the prevalence of COPD according to the Global Burden of Disease Study was estimated to be 11.6/1,000 in men and 8.77/1,000 in women (3), although COPD prevalence is higher in older adults and in countries where cigarette smoking has been, or still is, very common. Numerous reports indicate that mortality due to COPD continues to increase, with the global proportion of deaths attributed to COPD predicted to increase by approximately 65% between 2002 and 2030 (1, 4). In comparison, mortality rates related to cardiovascular disease (CVD) appear to be slowing, or even declining, in Western populations (4–7). This is due, at least in part, to more widespread use of therapies for CVD that have been shown to reduce mortality. One of the main contributing factors is increased uptake of the 3-hydroxy-3-methyl-glutaryl-coenzyme A (HMG-CoA) reductase inhibitors, or statins, for the treatment of dyslipidaemia and atherosclerotic disease (8–11).

Increased awareness and implementation of treatment guidelines may also contribute to reductions in CVD mortality, with physicians and patients becoming better educated about treatment goals and effective treatments, leading to increased uptake of statins and other cardiovascular medications. In addition, lifestyle modification programmes promoting improved awareness and better control of risk factors for CVD, such as reductions in smoking, blood pressure and cholesterol, have proved effective and may help to explain the slowing of CVD mortality observed in Western populations (6, 7).

This article considers the extent to which these insights from managing heart disease may be applicable to COPD, and whether currently available treatments for COPD can have a significant impact on mortality. For example, smoking cessation and long-term oxygen therapy (where indicated) have been shown to reduce mortality in randomized clinical studies (12–14). In addition, this review discusses the relationship between COPD mortality and key risk factors including airflow obstruction, the BODE index (incorporating body mass index, airflow obstruction, dyspnoea and exercise capacity), impaired health status, increased exacerbations, declining lung function and co-morbidities where systemic inflammation may provide the link between COPD and CVD (2, 15–18).

Studies have indicated the potential benefits of COPD treatments such as the inhaled corticosteroids (ICS), with or without long-acting β_2_-agonists (LABAs), on mortality in patients with COPD (19–32). The article also discusses the role of combination therapy with ICS/LABA (budesonide/formoterol or salmeterol/fluticasone propionate) in reducing exacerbations and improving health status, and their potential for decreasing COPD mortality. These benefits are examined from a similar perspective to that used to assess treatment outcomes in CVD (25–32).

### Trends in COPD and CVD mortality

COPD is widely acknowledged as a major, and increasing, cause of mortality and morbidity across the globe. It is currently the fourth leading cause of death worldwide (1). Causes of death in COPD patients include COPD-related deaths as well as other causes, for example, CVD and lung cancer (33, 34). COPD death rates increase with age, and are higher in males than in females (35). Globally, COPD accounted for 4.8% of deaths in 2002, a figure that the World Health Organization (WHO) predicts will increase to 7.9% by 2030 (4). COPD also has a significant impact on quality of life, as evidenced by WHO figures estimating a change in ranking for COPD in terms of the proportion of total disability-adjusted life-years lost, from rank eleventh in 2002 to rank fourth in 2030 (4).

Furthermore, there is evidence that the burden of COPD may be underestimated. Firstly, the disease is usually not diagnosed until it is clinically apparent and moderately advanced, while the existence of varying definitions of COPD have made it difficult to quantify the burden of the disease (36). Secondly, mortality data may substantially underestimate COPD as a cause of death by citing COPD as a contributory, rather than an underlying, cause of death, or by not citing COPD at all on death certificates. For example, in a study analysing the epidemiology of deaths in England and Wales, only 60% of deaths where obstructive lung disease (COPD or asthma) was mentioned on the death certificate were attributed to COPD/asthma as the underlying cause of death (34). In contrast, 94% of deaths mentioning MI on the death certificate were attributed to MI as the underlying cause of death (34). Similarly, data from Denmark and the USA, respectively, suggest that COPD is under-reported on death certificates (37) and that mortality related to obstructive lung disease is underestimated in studies that look only at the underlying cause of death (38). In these studies, the principal causes of mortality in COPD included CVD and lung cancer (37, 38).

There is evidence that COPD mortality may be worsening relative to mortality due to CVD, MI or stroke. For example, although WHO estimates suggest that ischaemic heart disease and stroke will remain ranked first and second leading causes of death worldwide in 2030, there is also evidence that the increase in CVD mortality is slowing substantially relative to COPD (4).

**Figure 1:**
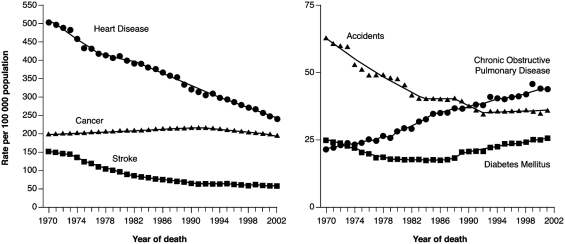
Trends in age-standardized death rates for the six leading causes of death in the United States, 1970–2002 (5). Reproduced with permission from JAMA 2005; 294: 1255–1259 Copyright © 2005 American Medical Association.

In fact, globally, the proportion of deaths attributed to COPD are predicted to increase by approximately 65% between 2002 and 2030, compared with less than 10% increases for both ischaemic heart disease and stroke (4). Furthermore, U.S. data examining trends in the leading causes of death (1) between 1970 and 2002 indicate a decline in mortality due to CVD, with age-standardized death rates decreasing by 52% for heart disease and 63% for stroke over this time period (Figure 1) (5). In contrast, age-standardized death rates due to COPD doubled between 1970 and 2002 (5). In the UK, deaths due to coronary heart disease (CHD) are also declining, with a 54% reduction in CHD mortality rates recorded between 1981 and 2000 (6).

**Figure 2:**
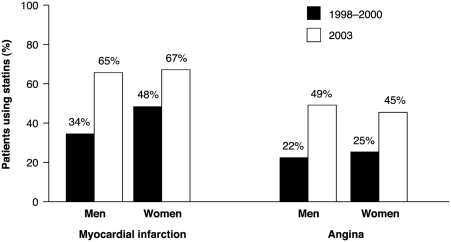
Prevalence of statin use for secondary prevention in patients with myocardial infarction or angina (8).

These trends in mortality may be explained by a number of factors. For example, almost 60% of the observed decrease in CHD deaths in the UK has been attributed to reduction in major risk factors, particularly smoking, while over 40% was attributed to the combined effects of adoption of modern cardiology treatments, including statins (6, 7). Of note, uptake of statins for the treatment of atherosclerosis has increased markedly over the past decade. This was illustrated in a questionnaire-based study conducted in the UK in 1998–2000 and 2003 in individuals with MI or angina (8). The study reported an almost doubling in statin usage during this time period (Figure 2) (8). In addition, increased awareness and implementation of treatment guidelines may also contribute to reductions in CVD mortality, with physicians and patients becoming better educated about treatment goals and effective treatments (39). Development of management guidelines for CVD has primarily been driven by findings from large clinical trials based on robust clinical outcomes (including mortality), resulting in increased prescribing in clinical practice (40).

In comparison with CHD, COPD is under-diagnosed and under-treated (36, 41). This may, at least in part, be due to the complexity and multicomponent nature of COPD, which comprises structural and functional changes occurring both inside and outside the lung. These include airflow limitation, airway inflammation, mucociliary dysfunction and structural changes in the airways and lung parenchyma, as well as systemic effects, such as skeletal muscle dysfunction, osteoporosis, cachexia, cardiovascular and nervous system abnormalities and chronic, low-grade inflammation (1, 2, 15, 18, 42–44). In addition, many individuals with COPD are not formally diagnosed because they consider breathlessness and limited exercise tolerance to be signs of ageing and regard their smoker's cough as normal (41). There is also evidence that, in contrast with asthma, COPD is under-treated by primary care physicians, particularly in the early stages of the disease (45). There is therefore a need for better understanding of the strongest predictors of mortality in COPD and the potential impact of therapies on COPD mortality.

### Predicting COPD mortality

There is considerable debate regarding the strongest predictors of mortality in COPD. Key predictors include advanced age (46–49), smoking status (48), low forced expiratory volume in 1 second (FEV_1_) (47, 48, 50), peak expiratory flow (50), low arterial partial pressure of oxygen (PaO_2_) (51, 52), low body mass index (52, 53) and reduced exercise capacity (49, 54). The BODE index – a composite index reflecting the multicomponent nature of COPD – has also proved to be a good predictor of mortality as it incorporates systemic as well as pulmonary characteristics of COPD (49, 55, 56).

Reduced health status/health-related quality of life is also gaining increasing recognition as a predictor of mortality. Instruments used to measure health status in COPD include the Medical Outcomes Study Short Form 36-item (SF-36) Health Survey, the St George's Respiratory Questionnaire (SGRQ), the Breathing Problems Questionnaire and the Seattle Obstructive Lung Disease Questionnaire. Health status measured using these questionnaires has been shown to predict mortality and, in some cases, hospitalization in COPD (54, 57–62). For example, the SGRQ provides an effective measure of health-related quality of life during acute exacerbations of COPD (63) and reliably predicts mortality in individuals with COPD (32, 54, 57–59, 61). Depression and disability also predict mortality in patients discharged from hospital following acute exacerbations of COPD (61).

There is growing interest in the impact of exacerbations on mortality and morbidity in COPD. Severe exacerbations of COPD have been shown to be associated with a worse prognosis, and mortality increases with the frequency of exacerbations (64). Exacerbations of COPD severe enough to require hospitalization have a significantly greater effect on mortality than those which can be managed in the community.

**Figure 3:**
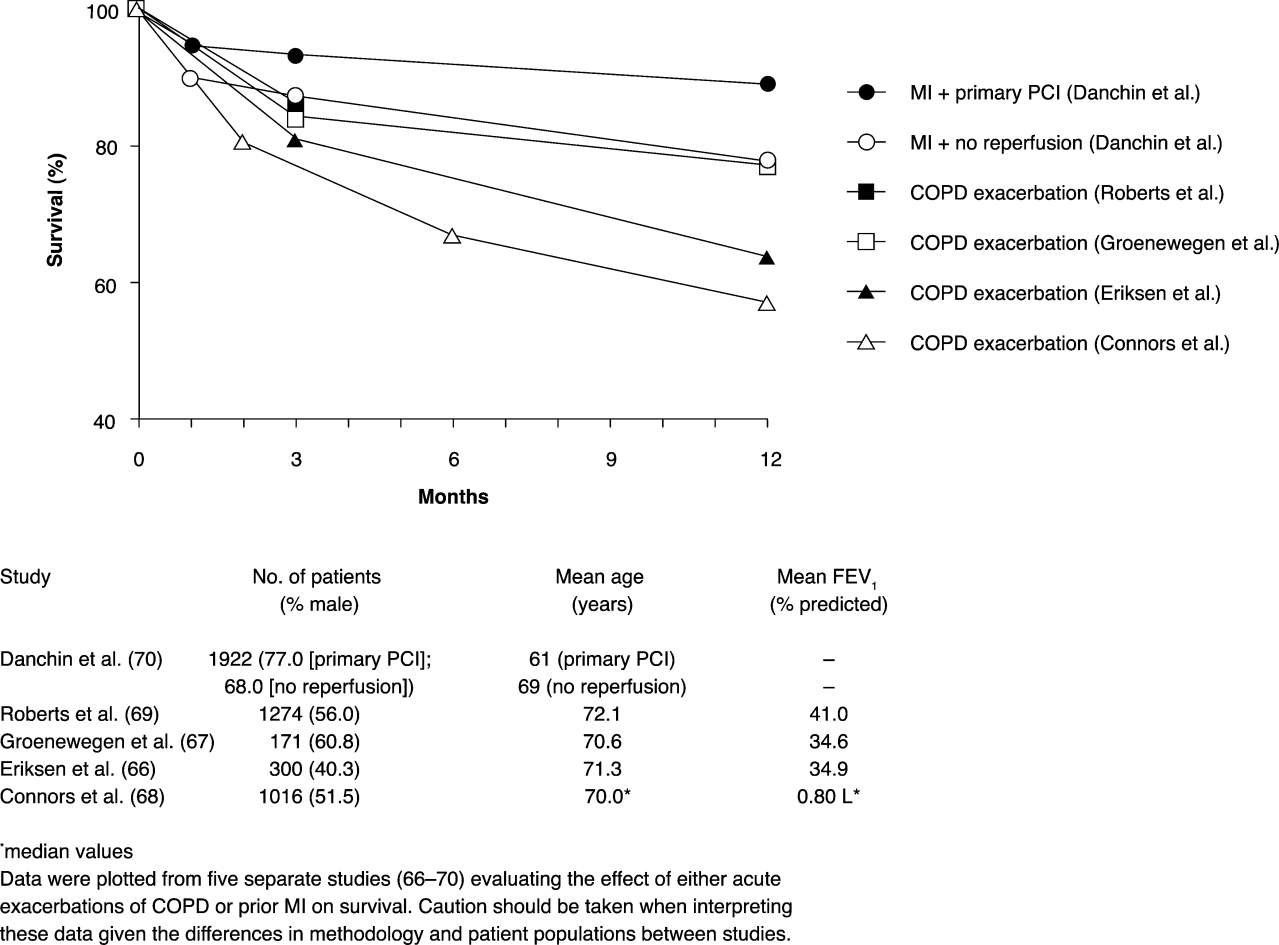
Effect of acute exacerbations of COPD versus myocardial infarction on survival (66–70). FEV_1_= forced expiratory volume in 1 second, PCI = percutaneous coronary intervention, MI = myocardial infarction, COPD = chronic obstructive pulmonary disease.

The magnitude of mortality following an exacerbation is not widely known. Data from several studies show that mortality at 12 months following hospitalization for an exacerbation of COPD is between 20% and 40%. This is much worse than the mortality observed following hospital admission with an acute MI, whether or not patients received acute reperfusion therapy (Figure 3) (32, 64–70). In addition, COPD exacerbations impair health-related quality of life and patient well-being, as well as contributing to a faster decline in lung function and increasing the need for hospital admissions (71–73). Given evidence that patients under-report exacerbations (71, 74), the extent of their effects on mortality and morbidity may be greater than current figures indicate.

In contrast, CVD risk factors are more clearly defined and more easily measurable. Epidemiological evidence has increasingly been used as a rationale for clinical trials in CVD, as reviewed in Ramsey et al. (40). This reflects recognition of the important role that managing risk factors plays in the clinical management of the disease. Several key risk factors have been directly linked to outcomes and epidemiological data have shown the importance of specific clusters of cardiovascular risk factors in certain individuals. For example, the INTERHEART study demonstrated that the nine traditional risk factors for CVD explained over 90% of the attributable population risk for MI (75). Cardiovascular risk scoring systems such as the Framingham risk calculator have been developed to identify individuals at particularly high risk of atherosclerosis and thus improve decision making about interventions for primary prevention of CVD.

Biomarkers such as cardiac troponins are also available to stratify risk and direct subsequent management of patients with acute events such as unstable angina or a suspected MI (76, 77). Incorporation of these findings into CVD treatment guidelines (76, 78, 79) has facilitated early diagnosis and allows physicians to optimize treatment.

Key biomarkers for CVD may also be related to exacerbations in COPD patients, for example, troponins are reported to be elevated in COPD patients suffering from acute exacerbations (80). However, despite increased interest in the association of inflammatory biomarkers (e.g., tumour necrosis factor alpha, leukotriene B4, interleukin-8 and biomarkers of oxidative stress) with exacerbations of COPD (1, 81), they do not provide information that can be used to stratify the risk of mortality or identify subgroups of patients requiring different management strategies.

### Impact of therapies on COPD mortality

The goals of effective COPD management include improving health status, preventing and treating exacerbations and reducing mortality (1). Control of risk factors, in particular the implementation of smoking cessation programmes, should have a significant impact on COPD prevalence, leading ultimately to a reduction in COPD mortality, as in the cardiovascular arena. As well as reducing the future prevalence of the disease, smoking cessation has been shown to reduce mortality in a clinical trial setting (12) and is the single most effective (and cost-effective) intervention to reduce the risk of developing COPD and stop its progression (1). However, the immediate benefits of stopping smoking in COPD may be less than those seen in CVD and patients with COPD may have particular difficulty quitting as it appears that smoking cessation strategies are often ineffective in individuals with COPD (82, 83).

COPD mortality is still continuing to increase and there is a need for pharmacological interventions that can reduce mortality in patients with established disease. Current pharmacological options for the treatment of COPD include short-acting β_2_-agonists (SABAs; e.g., salbutamol and terbutaline), LABAs (e.g., formoterol and salmeterol), short-acting and long-acting anticholinergics (e.g., ipratropium bromide and tiotropium bromide, respectively), theophylline, ICS (e.g., beclomethasone dipropionate, budesonide and fluticasone propionate), fixed-dose combinations of ICS/LABA and SABA/anticholinergic, and systemic glucocorticosteroids. Historically, the focus of these treatments has been on improving physiological measures of lung function and providing symptom relief, but there is now increasing interest in their impact on health status and their possible effects on reducing premature death. Of these treatments the most promising regarding reductions in mortality appear to be ICS, particularly in combination with a LABA.

*ICS and their impact on exacerbations, health status and mortality.* Until recently there was a lack of randomized clinical trial data on the effects of ICS on mortality and the only information available came from several retrospective/observational studies, which suggested that ICS may have a beneficial effect on mortality in patients with COPD.

Several population-based cohort studies evaluating longitudinal healthcare databases in Canada (19–21, 24, 84) have suggested that receiving ICS following hospitalization for COPD reduces the risk of death, but these studies have been criticized on the grounds that they include an “immortal time bias” which favours patients prescribed ICS (85).

The Canadian database study (19) is susceptible to immortal time bias as patients were classified as receiving ICS if they were dispensed a prescription within 90 days of discharge from hospital. Thus, in order to be eligible to enter the ICS group they must have survived at least as long as the interval from hospital discharge (which was defined as the cohort entry time) until ICS were prescribed and during this time they were effectively immortal as they could not die, whereas patients not in the ICS group could die at any time from cohort entry. Whether or not this time period affected the analysis is not known, but the potential for the bias to influence the results must be considered. It has also been proposed that immortal time bias may also affect the hierarchical cohort design used in the study by Soriano and colleagues (20). In this case the criticism centres around the exclusion of immortal time in the non-ICS group and the impact this may have had on the survival analysis. In this analysis patients who were not receiving ICS at cohort entry but who received them at some point during the follow-up period were excluded from the follow-up analysis. It has been suggested that excluding these patients from the follow-up analysis led the authors to ignore the immortal time these patients experienced prior to receiving ICS and led to a flawed conclusion about the effect of ICS on mortality (86). On the other hand, it seems very reasonable to base the conclusions on analysis of survival in two cohorts, one of which received ICS at cohort entry and another in which patients did not receive ICS throughout the follow-up period.

The epidemiological data on survival benefits of ICS is supported by observational data in very severe COPD patients with respiratory insufficiency who were put on long-term oxygen therapy, compared to those who did not use ICS concomitantly (22). However, it is important to note that these are observational data, that patients were not randomized to steroid therapy, and that little or no information is available to assess baseline risk in the treatment groups.

Further insight into the impact of ICS on outcomes is provided by data from prospective randomized clinical trials, including data from pooled trials. For example, the Inhaled Steroids in Obstructive Lung Disease (ISOLDE) trial was a double-blind, placebo-controlled study investigating the impact of ICS in 751 patients with moderate to severe COPD (87). In ISOLDE, fluticasone propionate significantly reduced the rate of exacerbations and slowed the rate of decline of health-related quality of life (87–89) and a *post hoc* analysis of the study showed a nonsignificant trend towards improved survival in patients from the fluticasone propionate arm (*P* = 0.069) (90). Although significance was not reached, this may be due to the small size of the study population and the limited follow-up interval. Similar findings come from a systematic review of randomized placebo-controlled ICS trials in COPD patients by Alsaeedi and colleagues (91): the summary relative risk of the trials was consistent with a small survival benefit, but the effect was not statistically significant (91).

Sin and colleagues carried out a pooled analysis of intention-to-treat data from seven large, long-term randomized trials, collectively involving more than 5,000 patients (23). This pooled analysis, known as the Inhaled Steroid Effects Evaluation in COPD (ISEEC) study, found that ICS reduced all-cause mortality by over 25% relative to placebo over a mean follow-up of 26 months (23). These beneficial effects appeared to be most pronounced in women and former smokers (23). Interestingly, in a retrospective analysis of the 3-year EUROSCOP study of patients with mild to moderate COPD, which was included in the ISEEC pooled analysis, the incidence of pre-defined cardioischaemic events was significantly lower in the budesonide arm compared with placebo (92). However, the authors note that additional studies are required to determine whether the survival benefits persist beyond 2–3 years, and to evaluate long-term adverse events (23).

Overall, there is a large body of evidence which suggests that ICS provide a survival benefit in COPD. However, data from the fluticasone arm in the TOwards a Revolution in COPD Health (TORCH) study, which investigated the impact of salmeterol/fluticasone propionate in a single inhaler on mortality and SGRQ over 3 years in over 6,000 patients with moderate to severe COPD, did not show a reduction in all-cause mortality at 3 years versus placebo (93). The reasons why this result differs from the observational studies *and post hoc* analyses are not clear, particularly as fluticasone propianate reduced the rate of exacerbations.

Although more episodes of pneumonia were reported as adverse events in patients treated with ICS there was no difference in the incidence of fatal pneumonia and it may simply be that when complete follow-up is available, biases are eliminated so that the null hypothesis that ICS reduce mortality is disproved. Further studies are needed to clarify this.

*Long-acting bronchodilator use in patients with COPD.* LABAs have an important role in managing the symptoms of COPD, with side effects generally considered to be predictable and dose-dependent. A recent meta-analysis of randomized controlled trials evaluating the use of LABAs over a period of at least 3 months has suggested an increased rate of respiratory deaths with LABAs compared with placebo (94). However, recent randomized studies have shown that LABAs are well tolerated in patients with COPD and have a good safety profile (95–97).

Similar concerns have been expressed with regard to LABA use in adults with asthma; however, it is clear that when used appropriately, i.e., in combination with ICS, there is no evidence of a safety risk (98, 99). Any observed adverse outcomes associated with LABA monotherapy were caused by “masking of inflammation” rather than a toxic effect of the drugs, and such concerns are of less relevance in COPD where the pattern of inflammation and the effects of ICS on this are different.

The recent prospective study by Gudmundsson et al. indicated an improvement in survival, in patients taking LABAs alone, among those who had been hospitalized for acute exacerbations of COPD, as described previously (32). More importantly, the TORCH study also supported the safety of LABAs in individuals with COPD, and showed a trend towards increased survival for patients treated with salmeterol alone compared with placebo (hazard ratio 0.879, 95% CI 0.729–1.061) and no increase in adverse events (93).

The long-acting muscarinic antagonist (LAMA) tiotropium also provides effective symptom control and reduces exacerbation frequency in patients with COPD (100). *Apost hoc* analysis of 1-year data suggests that it may reduce the rate of decline of FEV_1_ (101), and, if this is a real effect, LAMAs may have an effect on mortality. The results of the UPLIFT trial (102) should help shed some light on this when they are published.

*The benefits of adding ICS to LABAs.* Combined therapy with ICS/LABA improves health status and reduces the frequency of exacerbations, as demonstrated by two 12-month randomized studies evaluating the effects of budesonide/formoterol in a single inhaler in individuals with COPD (29, 30). In the first study (Szafranski et al.), budesonide/formoterol reduced the mean number of severe exacerbations (defined as requiring oral corticosteroids and/or antibiotics and/or hospitalization due to respiratory symptoms) by 24% versus placebo and 23% versus formoterol in 812 patients with COPD and significantly improved health-related quality of life versus placebo (29).

In the second study (Calverley et al.), of 1,022 patients with COPD, those taking budesonide/formoterol had a prolonged time to first exacerbation requiring medical intervention (defined as requiring oral corticosteroids and/or antibiotics or hospitalization), fewer exacerbations requiring medical intervention and a clinically relevant improvement in health status (measured with the SGRQ) compared with those on placebo (30).

Similarly, in the TRial of Inhaled STeroids ANd long-acting beta-agonists (TRISTAN) study, use of salmeterol/fluticasone propionate in a single inhaler reduced the frequency of exacerbations (defined as requiring oral corticosteroids and/or antibiotics) in individuals with COPD (103). These results have also been confirmed in the TORCH 3-year outcomes study, as described in more detail next (93).

Data from number needed to treat (NNT) analysis also demonstrate the beneficial effects of ICS/LABA in preventing severe COPD exacerbations (31). NNT analysis is a useful method because it allows physicians to easily quantify the benefits of alternative treatment options on disease outcomes in terms of the number of patients who need to be treated in order to see a particular outcome (31, 104, 105). Using data from the trials mentioned earlier (29, 30), NNT analysis showed that 2.1–2.4 patients would need to be treated with budesonide/formoterol in a single inhaler compared with formoterol alone (or placebo) in order to prevent one exacerbation requiring medical intervention in 1 year (31). In other words, treating 100 patients with severe to very severe COPD with budesonide/formoterol versus formoterol alone (or placebo) would prevent 42–47 exacerbations requiring medical intervention during 1 year of treatment (31). In the recent TORCH study, the NNT for salmeterol/fluticasone propionate in reducing exacerbations was 8.3 versus salmeterol and 3.6 versus placebo, as calculated from the annualized exacerbation rates (106).

Using data from the TRISTAN study, the NNT for the prevention of exacerbations by salmeterol/fluticasone propionate in a single inhaler over 1 year in a similar patient population was of similar magnitude (3.0 versus placebo). In a separate trial, the VIVACE study, which compared the impact of salmeterol/fluticasone propionate and salmeterol on exacerbations in individuals with severe COPD, the NNT to prevent one moderate/severe exacerbation per year with salmeterol/fluticasone propionate was 2.08 versus salmeterol alone (107).

As is the case for ICS, until recently there was virtually no published data from prospective studies on the impact of ICS/LABA on survival in patients with COPD. However, other data suggest that combination therapy may have an effect on mortality, for example, retrospective data suggest that ICS/LABA may improve survival versus either ICS or LABA alone (20, 25, 26). In an observational study analysing data from the UK General Practice Research Database, COPD patients treated with fluticasone propionate and salmeterol (n = 1,045) were compared with those who regularly used other bronchodilators but not ICS or LABAs (n = 3,620) (20). After 3 years, survival was significantly greater in fluticasone propionate and/or salmeterol users versus the reference group, with the highest survival advantage found in users of combined salmeterol/fluticasone propionate (20).

Mortality decreased as the number of prescriptions of fluticasone propionate and/or salmeterol increased (20). A separate retrospective cohort analysis of the UK General Practice Research Database compared rehospitalization for a COPD-related condition or death within a year of first hospitalization in patients prescribed ICS and/or LABA (n = 3,636) versus reference patients, who were on SABA only (n = 627) (25).

Use of ICS with or without LABAs was associated with a reduction in rehospitalization or death in COPD patients, with a 41% risk reduction in users of ICS/LABA (*P* < 0.05), 16% reduction in ICS users (*P* < 0.05) and 10% reduction in LABA users (nonsignificant) versus the reference group (25). In another retrospective cohort study, this time of two U.S. managed care organizations, COPD patients who used ICS, either alone or in combination with LABAs, were again shown to have substantially improved survival, even after adjustment for asthma and other confounding factors (26).

Data from a prospective study (Gudmundsson et al.) have recently confirmed the findings of these retrospective analyses, showing an association between ICS and LABA treatment and reduced mortality (32). Four-hundred and sixteen patients with COPD who had been hospitalized for an acute exacerbation were followed for 2 years (32). During the follow-up, 29% of the patients died, with diabetes, advanced age, low FEV_1_ and lower health status associated with an increased risk of mortality (32). Patients treated with ICS and/or LABAs had a lower risk of death compared with patients taking neither of these treatments (32); however, patients were not randomized to treatment and thus considerable potential for bias exists.

**Figure 4:**
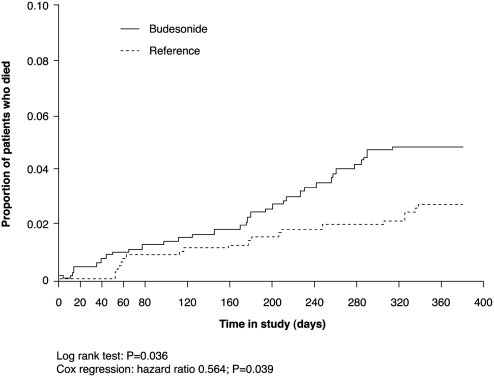
Effect of budesonide-containing therapy (budesonide/formoterol or budesonide) versus reference therapy (formoterol or placebo) on mortality (27).

A recent pooled analysis of two large randomized studies has further investigated the impact of adding ICS to bronchodilators on COPD mortality (27). Mortality and baseline data were pooled from the Szafranski et al. and Calverley et al. studies (29, 30), together involving more than 1,800 individuals with COPD who were treated with budesonide/formoterol (in a single inhaler) or budesonide versus formoterol or placebo over 1 year. The pooled data indicated that budesonide, either alone or in combination with formoterol, significantly reduces the risk of death from any cause at 1 year compared with formoterol or placebo maintenance therapy (*P* = 0.039; Figure 4) (27). Effects of budesonide on survival were apparent in all patients, irrespective of age, sex or lung function (FEV_1_) (27).

All these analyses suffer from the same problems as the observational studies of ICS discussed above. The TORCH study was designed to provide prospective data on this question in a 3-year randomized clinical trial. This study differed from previous studies by specifying all-cause mortality as a primary endpoint and including rigorous methods to establish whether all patients, including those that had dropped out, were alive or dead at the end of 3 years (93, 108). Results from TORCH reported that whilst salmeterol/fluticasone propionate decreased the risk of mortality over the 3 years by 17.5% compared with placebo, the reduction did not quite achieve statistical significance (*P* = 0.052) (93).

Calverley and colleagues plausibly suggest the study failed to reach statistical significance because it may have been underpowered (93). TORCH was designed to have 90% power to detect an effect of 4.3 percentage points on overall mortality, while the final results showed a reduction of 2.6 percentage points. A prespecified secondary analysis (Cox proportional-hazards testing) showed a significant reduction in the hazard ratio to 0.811 (95% CI 0.670–0.982; *P* = 0.03), suggesting that the effect on mortality is genuine. The results of the study are also likely to represent a conservative estimate of the effect of combination treatment on mortality as a result of the inherent limitations associated with a 3-year study which includes a placebo arm. This may have led to bias due to early and differentiated drop-out of patients (109). For example, an early and larger drop-out rate in the placebo group may generate a healthy survivor bias for those staying on in the study. Additionally, some of the patients who have dropped out of the placebo arm may subsequently have received active treatment from their clinicians. Thus, when analysed on an intention-to-treat basis, such patients would lead to an underestimation of the benefits of combination therapy.

TORCH also demonstrated that salmeterol/fluticasone propionate improved lung function and health status (measured using the SGRQ) (93). Salmeterol/fluticasone propionate also reduced moderate/severe exacerbations, with significantly superior effects observed compared with placebo, fluticasone propionate alone and salmeterol alone over 3 years (93). This reduction in exacerbations was of similar magnitude to that reported in the TRISTAN study (103). Data from TORCH also indicated that salmeterol/fluticasone propionate was well tolerated, with a favourable long-term benefit/risk ratio compared with placebo (110).

An unexpected but statistically significant increase in the incidence of non-fatal pneumonia was observed in patients taking salmeterol/fluticasone propionate compared with placebo in the TORCH study (3-year duration) (93, 110). This concords with data from the VIVACE study (2-year duration), which showed an increase in pneumonia in patients taking salmeterol/fluticasone propionate versus salmeterol alone (107). Similar findings were not seen in the Calverley and Szafranski studies of budesonide/formoterol (1-year duration): pneumonia incidence was less than 5% and was similar between the budesonide and non-budesonide groups (29, 30).

It is not clear why there is a discrepancy between the results of the TORCH study (93) and previous studies (20, 23, 25–27, 32). One reason may be that the observed differences are due to variations in patient populations. Another is that complete information was available on vital status in all randomized patients in the TORCH study, whereas it was only available in those who completed the previous studies and thus these may have been influenced by a healthy survivor effect. Additional randomized, controlled clinical trials are required to investigate further the effects of ICS/LABA on mortality.

*Additional benefits of statins.* Interestingly, statins may provide additional benefits in patients with COPD. Retrospective data demonstrate that, in patients who had been hospitalized for COPD exacerbations, treatment with statins or ICS post-discharge led to significant reductions in mortality, with the greatest benefits reported in patients taking both statins and ICS (111, 112). In another retrospective study, both cardiovascular and pulmonary outcomes were reduced in COPD patients taking statins, angiotensin-converting enzyme inhibitors and angiotensin receptor blockers (113). Use of these agents was associated with reduction in COPD hospitalization and total mortality, not only in the high cardiovascular risk cohort but also in the low cardiovascular risk cohort, while the combination also reduced MI in the high cardiovascular risk cohort (113). A further retrospective study conducted by Younis and colleagues reported a 35% decline (*P* = 0.02) in the rate of hospitalization and emergency room treatment among COPD patients (114). Randomized clinical trials are required to confirm these observations and to investigate the mechanism by which these agents may exert their effects in individuals with COPD.

*Limitations of randomized controlled trials, observational studies and post hoc analyses.* When considering the studies discussed above it is important to note that, with the exception of the TORCH study, the studies were not designed to assess mortality rates. Patients in the database studies were not randomized to the different treatment arms and in both these and the *post hoc* analyses, intention-to-treat analyses are not possible in most cases, as patients are not followed after study discontinuation or biases such as immortal time bias are introduced.

In most studies entry criteria excluded patients at higher risk of death, e.g., those with concomitant CVD and recent exacerbations, and most studies were underpowered as death was an uncommon event. All studies were of relatively short duration and significant numbers of patients withdrew from the randomized controlled trials. Nevertheless, taken together, these studies provide a compelling case that pharmacotherapy can affect mortality in COPD.

*Looking forward: what can we learn from CVD?* In tandem with more effective treatments for acute MI, better control of risk factors for CVD, notably using statins, has had a marked impact in decreasing mortality due to CVD. Large-scale clinical trials, including the Scandinavian Simvastatin Survival Study (4S), the West of Scotland Coronary Prevention Study (WOSCOPS) and the Aggressive Lipid-Lowering Initiation Abates New Cardiac Events (ALLIANCE) study (9–11), have demonstrated the beneficial effects of statin therapy on survival, and key findings from these studies have been incorporated into CVD treatment guidelines. Increased awareness and implementation of guidelines has resulted in physicians and patients becoming better educated about treatment goals and effective treatments, leading to increased prescribing of statins and other cardiovascular medications that improve survival. This in turn has contributed to the deceleration, or even decline, in CVD mortality rates observed in Western populations (4–8).

What can we do to decrease deaths due to COPD? Once viewed as an irreversible condition, COPD is now considered a treatable disease. As with CVD, improved control of risk factors for COPD (i.e., smoking cessation) will, over time, have a major impact on mortality. Pulmonary rehabilitation and long-term oxygen therapy in hypoxic patients can improve survival, while lung volume reduction surgery is also beneficial in selected cases. However, for many patients with established disease the major impact on mortality is likely to come from pharmacological interventions which mirror the benefits of statins in heart disease. Much effort has been put into developing such treatments, but we may already have drugs that can reduce mortality.

**Figure 5:**
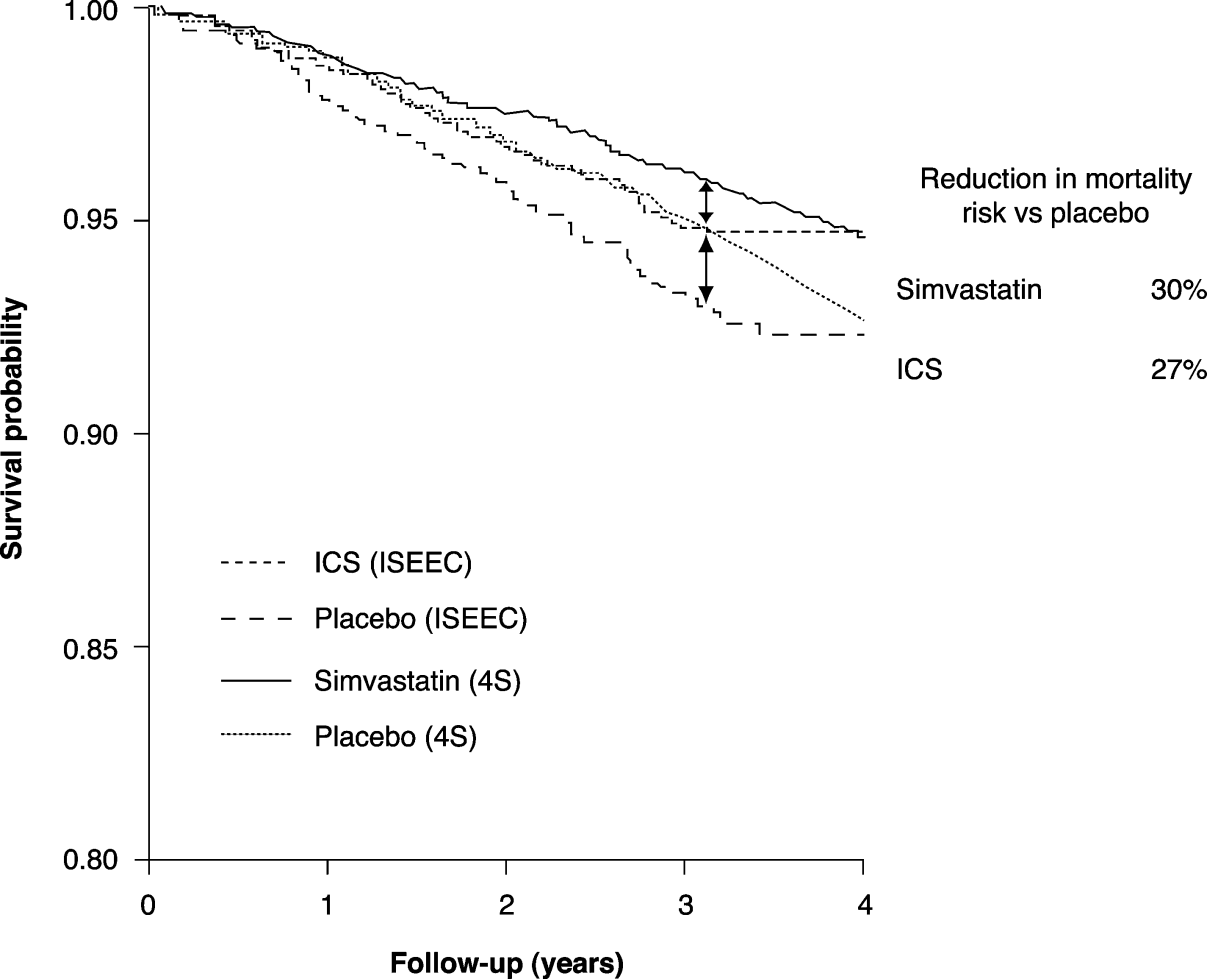
Survival benefit observed with ICS in COPD compared with that of the statins in coronary heart disease (9, 23). ICS = inhaled corticosteroids, ISEEC = Inhaled Steroid Effects Evaluation in COPD, 4S = Scandinavian Simvastatin Survival Study.

As discussed before, the use of ICS, either alone or in combination with LABAs, appears to have a beneficial effect on survival which may be comparable to the effects seen with the statins. Figure 5 compares data from one of the key cholesterol-lowering trials in patients with CHD, 4S, with findings of the ISEEC study in patients with COPD (9, 23). Furthermore, statins also have anti-inflammatory properties (115, 116), in addition to their lipid-lowering properties, which may have a beneficial impact in COPD (112, 113, 117). Currently, statins are only indicated in those suffering from hyperlipidaemia; however, further research is needed to explore the impact of statin therapy in COPD patients, and specifically, this research should focus on the impact of statins alone as well as the effects of statins given concurrently with ICS/LABA combination therapy.

NNT analysis provides a further indication of the fact that we may have overlooked the efficacy of the drugs we already have available. The impact of ICS on survival in COPD compares very favourably with other commonly accepted treatments for chronic diseases (28, 31). For example, using data from the Ontario population-based cohort study described earlier (19), the NNT for ICS in COPD has been calculated for survival (NNT = 27), comparing favourably with the NNT for ICS in asthma (NNT = 72) and for the commonly used fibrate, gemfibrozil, in coronary artery disease (NNT = 1,000) (28). Note that these estimates are from studies with very different designs and which are, therefore, subject to a number of systematic biases (28). Other sample NNT values for preventative interventions are shown in Table 1. Again, note that these data should be interpreted with caution because of the limitations of comparing NNTs across different drugs/diseases. For this reason, physicians are encouraged to use NNT data in conjunction with other considerations when making clinical decisions. For example, NNT values can vary widely even for the same drug/disease according to factors such as baseline disease severity and other differences in study populations (31). Hence it is important to compare ‘like with like’ with regard to patient populations, study designs and outcome endpoints. In addition, for some patients a non-fatal MI or stroke may be a more significant event than a COPD exacerbation, with greater consequences for impact on future mortality and health status. However, the impact of a COPD exacerbation has often been underestimated in the past, so these comparisons are still relevant (31).

**Table 1:** Examples of NNTs for different drug classes

**Drug class**	**Trial**	**NNT**
Lipid-lowering drugs	Meta-analysis (118)	69 for primary prevention of non-fatal MI or cardiovascular death
Aspirin	European Stroke Prevention Study (119)	34 for secondary stroke prevention
ACE inhibitors	HOPE Trial (120)	27 for cardiovascular events or death
Statins	4S Trial (121)	17 for cardiovascular events, 30 for death
Salmeterol/fluticasone propionate	GOAL Study (122)	10–20 for severe asthma exacerbation
Budesonide/formoterol	Halpin et al. (31)	2.1–2.4 for COPD exacerbation requiring medical intervention
Salmeterol/fluticasone propionate	VIVACE Study (107)	2.08 for moderate/severe exacerbation

ACE = angiotensin-converting enzyme; 4S = Scandinavian Simvastatin Survival Study; HOPE = Heart Outcomes Prevention Evaluation; GOAL = Gaining Optimal Asthma ControL.

## CONCLUSION

COPD is a major cause of death worldwide. Action is needed to improve understanding of factors that can be incorporated into clinical practice to identify patients at risk of dying from their COPD and to increase the number of patients treated for COPD. Increased use of therapies such as ICS, particularly combined with LABAs, has shown promising effects in decreasing mortality in patients with COPD, as well as reducing exacerbations and improving health status. In concert with better control of risk factors for COPD, in particular smoking, these therapies may have the potential to help reduce death rates from COPD and to improve the outlook for patients with COPD.

The concept of secondary prevention following an exacerbation needs to be introduced more widely. Patients who have had an exacerbation should be offered treatment with combinations of drugs which have been shown to reduce the risk of future exacerbations (i.e., they should be prescribed combinations of ICS, LABA and LAMA). In addition, COPD patients should be encouraged to address lifestyle factors such as stopping smoking and controlling weight and they should undertake pulmonary rehabilitation when indicated. If used appropriately these treatments may lead to reductions in COPD mortality, mirroring the significant slowing of CHD mortality seen over the last few decades. Furthermore, combining COPD therapy with other drugs such as statins may be beneficial in targeting systemic inflammation, an underlying characteristic common to both COPD and CHD, offering the potential to further influence disease progression.

We may already have the tools to improve survival in COPD, but they are frequently underused and we must learn lessons from the management of CVD and copy their success if we are to make a real difference to patients.
